# Relapsing polychondritis in systemic sclerosis: A rare vasculitic mimic

**DOI:** 10.1177/23971983221141599

**Published:** 2023-01-09

**Authors:** Carolina Teles, Chiranthi Kongala Liyanage, Geoffrey Chow, Christopher P Denton, Voon Ong

**Affiliations:** 1 Department of Internal Medicine, Centro Hospitalar e Universitário de Coimbra, Coimbra, Portugal; 2 Faculdade de Medicina, Universidade de Coimbra, Coimbra, Portugal; 3 Centre for Rheumatology, Royal Free Hospital London, London, UK; 4 Department of Pharmacology, Faculty of Medicine, University of Colombo, Colombo, Sri Lanka; 5 Department of Imaging, Royal Free Hospital London, London, UK; 6 Centre for Rheumatology, Division of Medicine, University College London, London, UK

**Keywords:** Systemic sclerosis, relapsing polychondritis, vasculitis, auricular chondritis, genetic predisposition

## Abstract

**Introduction:**

Relapsing polychondritis is a rare, immune-mediated disease characterised by inflammation of cartilaginous structures. Auricular chondritis, sparing the fatty lobule, is the most typical feature, followed by nose and laryngotracheal involvement. Albeit rare, neurologic involvement is reported with relapsing polychondritis. Cranial nerve involvement is the most frequent neurologic manifestation and is probably due to an underlying vasculitic process. Approximately one-third of relapsing polychondritis patients can overlap with other systemic diseases, including other autoimmune connective tissue diseases, but association with systemic sclerosis has very rarely been described.

**Case description:**

A 63-year-old woman presented with acute new-onset severe dysphagia, accompanied by hoarseness and preceded by pain, swelling and erythema of the left pinna, unresponsive to antibiotics. She had a history of long-standing limited cutaneous systemic sclerosis. Cranial nerve examination revealed right-sided palatal palsy, and left vocal cord palsy was found on fibreoptic nasendoscopy. Magnetic resonance imaging of the head and neck showed bilateral enhancement of an extracranial segment of the glossopharyngeal and vagus nerves. Clinical features and imaging findings were consistent with relapsing polychondritis, which successfully responded to high-dose steroids.

**Conclusions:**

This is a case of relapsing polychondritis mimicking progression of systemic sclerosis, showcasing its challenging features. It emphasises the importance of early diagnosis and prompt management with potential impact on the outcome, while highlighting the complex interplay between these two disease entities and vasculitic mechanisms, which may reflect the shared network of genetic predisposition across autoimmune rheumatic diseases.

## Introduction

Relapsing polychondritis (RPC) is a rare, episodic, inflammatory and immune-mediated condition, affecting the cartilaginous and proteoglycan-rich tissues, particularly of the ear, nose, joints and respiratory tract.^1^–^5^ Its aetiology is largely unknown.^1^–^5^ It can present with a wide range of clinical manifestations, of which auricular chondritis is the commonest feature.^2^–^4^ RPC can also involve other structures, such as the eyes, blood vessels and cardiac valves, and some manifestations such as central nervous system (CNS) involvement can be the result of an ensuing vasculitic process.^2^–^4^ In about 30% of the patients, RPC can overlap with other diseases, including autoimmune diseases and hematologic disorders.^3^–^6^ RPC in the setting of systemic sclerosis (SSc) has very rarely been described.^7,8^

## Case description

**Figure 1.**
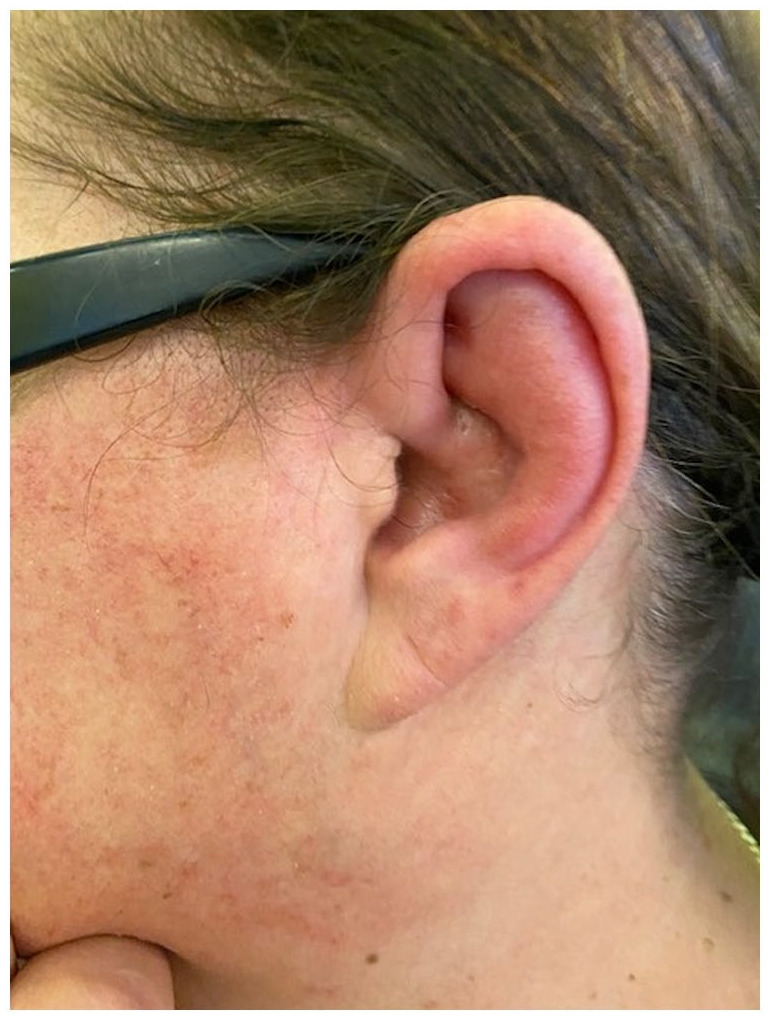
Left auricular chondritis, sparing the fatty lobule.

A 63-year-old woman presented with acute-onset severe dysphagia, for both solids and liquids, with inability to swallow saliva, progressing over the course of 1 week, and ultimately requiring nasogastric tube (NGT) insertion. The dysphagia was accompanied by worsening hoarseness of voice, and it was not associated with odynophagia, stridor, or throat or neck pain. Three days earlier, she developed left pinna pain, which progressed to swelling and erythema, sparing the lobule, and did not respond to amoxicillin. She had also noted mild blurring of vision without eye pain or redness. She did not complain of hearing loss, vertigo, headache, photophobia or other neurologic symptoms. There were no associated constitutional or joint symptoms. No preceding triggers were identified. She had long-standing limited cutaneous SSc, with positive antinuclear antibody (ANA) and negative extractable nuclear antigens (ENAs) with no specific SSc-hallmark antibodies, presenting with Raynaud’s phenomenon for 10 years, sclerodactyly and puffy fingers for 7 years, gastric acid reflux and history of polyarthralgia. Annual screening with echocardiogram and lung function has been reassuring. Her current medication was hydroxychloroquine 200 mg twice daily and losartan 25 mg once daily. Examination revealed an erythematous, swollen left pinna, sparing the lobule (Figure 1), with no tragus or mastoid tenderness. Neurologic examination revealed a right-sided palatal palsy, but was otherwise unremarkable.

**Figure 2.**
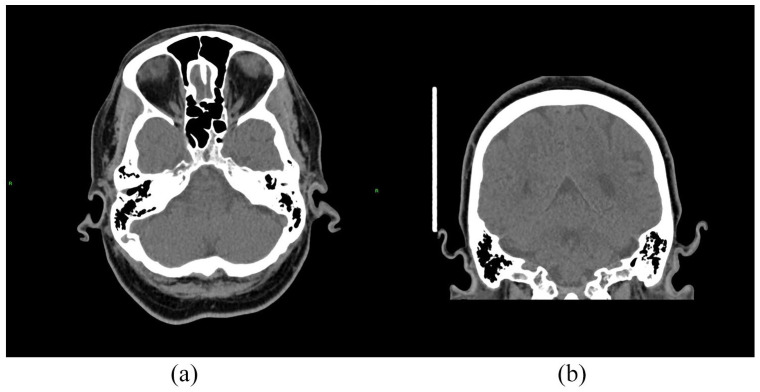
Thickening of the left pinna on head CT: (a) axial and (b) coronal.

**Figure 3.**
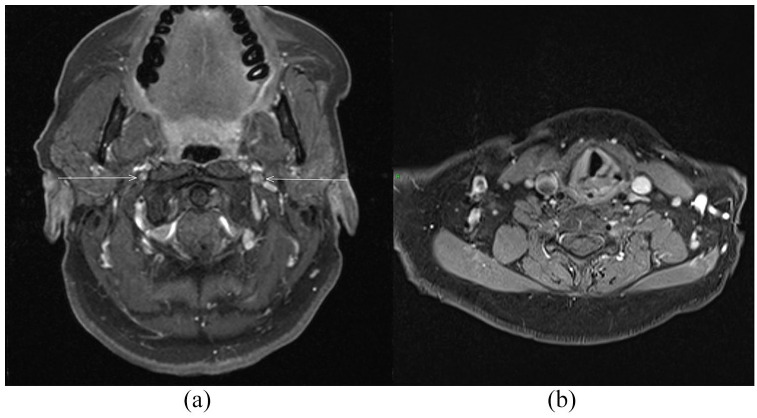
(a) Bilateral thickening enhancement of the glossopharyngeal and vagus nerves (arrows) on MRI scan (axial T1-weighted fat saturated post contrast sequence). (b) Left vocal cord palsy seen on MRI scan (axial T1-weighted fat saturated post contrast sequence).

Laboratory investigations showed raised C-reactive protein of 21 mg/L (range, 0–5), which was previously normal, and normal renal and liver function with normal urine analysis. ANA was positive (titre 1:1280), with negative ENAs and ds-DNA antibody. Cyclic citrullinated peptide antibody (CCP Ab), rheumatoid factor and anti-neutrophil cytoplasmic antibodies (ANCA) were also negative. Complements C3 and C4 were normal. HIV, hepatitis B virus (HBV), hepatitis C virus (HCV), Influenza A and B, respiratory syncytial virus (RSV) and SARS-CoV-2 were negative. Fibreoptic nasendoscopy showed left vocal cord (VC) palsy. Computed tomography (CT), axial and coronal, scan of the head showed thickening of the left pinna (Figure 2). Magnetic resonance imaging (MRI) of the head and neck (Figure 3) revealed bilateral thickening enhancement of the glossopharyngeal and vagus nerve complexes, just below the jugular foramen (Figure 3(a), arrows), as well as signs of left VC palsy, with enlargement of the left laryngeal ventricle and pyriform fossa, thickening of the left aryepiglottic fold and antero-medialisation of the left arytenoid cartilage (Figure 3(b)). Intracranial pathology was excluded. CT of neck-chest-abdomen-pelvis excluded malignancy and laryngotracheal compression. Transthoracic echocardiogram was unremarkable, with no evidence of valvulochondritis.

The diagnosis of RPC was made clinically supported by the imaging findings. Although this case did not completely fulfil the diagnostic criteria for RPC,^2,3,5^ the rapid progression with vasculitic involvement of the ninth and tenth cranial nerves, presenting with auricular chondritis, severe dysphagia and voice hoarseness, necessitated immediate treatment. Patient received pulses of intravenous (IV) methylprednisolone 500 mg daily for 3 days, followed by IV hydrocortisone 40 mg four times a day, later switched to oral prednisolone 40 mg daily. Two days after initiating treatment, patient noticed improvement of the pinna erythema, with full resolution in a few days. Blurring of vision also completely resolved in a few days. The dysphagia improved gradually, and NGT was removed on day 15 with the patient being able to swallow liquid and solids, including tablets. Given the rapid progression with CNS involvement, IV cyclophosphamide 12.5 mg/kg/pulse (dose adjusted according to age) was initiated with steroid tapering.

## Conclusion

RPC is a rare, episodic, immune-mediated disease, characterised by progressive and recurrent inflammation of cartilaginous tissues.^1^–^5^ In about one-third of the patients, RPC is associated with other diseases, such as autoimmune diseases, other rheumatologic diseases and malignancy.^3^–^6^ Association with SSc has very rarely been described.^7,8^ The exact aetiology of RPC is unknown, but is most likely multifactorial, resulting from a combination of genetic susceptibility with involvement of both humoral and cell-mediated immune responses, including cartilage-specific autoimmunity with circulating autoantibodies against collagen proteins II, IX and XI.^2^–^4^ RPC can present with a variety and often non-specific symptoms, affecting several organs, most commonly the ears, nose, eyes, laryngotracheal tract and joints.^1,3^–^5^ Auricular chondritis, sparing the lobule, is the most typical manifestation, occurring in over 80% of the cases.^1^–^5^ Laryngeal chondritis affects about 50% of the patients, presenting with cough, hoarseness and localised pain.^1^–^5^ Ocular manifestations, often due to episcleritis, scleritis or keratitis, occur in 20% to 60% of patients and are frequently mild.^1,2,4^ Neurologic involvement in RPC is rare and most commonly affects the cranial nerves, namely the second, sixth, seventh and eighth,^2,4,5^ although CNS manifestations, including encephalitis, myelitis and dementia, have also been reported.^2^–^6^ Both peripheral and CNS involvement may be the result of a vasculitic process, which may be an underlying mechanism of RPC pathogenesis.^2^–^4^ In fact, histologically proven vasculitis has been reported with RPC, and it may involve small, medium and large vessels, affecting organs ranging from the skin and kidney microvasculature to the aorta.^2,5,6,9^ It has also been reported that activation of effector T cells in the lungs may cross the blood–brain barrier leading to the proinflammatory state within the CNS, and this mechanism may link the inflammatory responses for both upper airway and CNS structures in RPC.^10^ RPC diagnosis is mainly clinical and there are no pathognomonic signs, but laboratory testing, imaging, including CT and MRI, and cartilage biopsy may be helpful and confirmatory.^1,2,5^ McAdam’s clinical diagnostic criteria were the first to be established, but were expanded by Damiani et al. to include histological confirmation or response to steroids, and later modified by Michet et al.^11–13^ SSc is also an uncommon immune-mediated connective tissue disease, which is characterised by fibrosis and vasculopathy, can involve multiple organs and present with variable clinical features.^14,15^ Overlap of SSc with other rheumatologic diseases is seen in up to 20% of the patients.^15^

We present a case of long-standing limited cutaneous SSc with a first presentation of RPC. The clinical and investigational features strongly suggest the diagnosis of RPC,^3^ since the patient presented with the highly typical auricular chondritis, with pinna pain, swelling and erythema, sparing the lobule, and with cranial nerve involvement, a known first manifestation of this disease,^16^ both of which rapidly and successfully responded to steroids. Furthermore, the lack of other cartilaginous involvement can be the result of an early diagnosis and treatment, since this was her first manifestation and disease progression may have been averted. On the other hand, we hypothesised that laryngeal cartilage and the contiguous left recurrent laryngeal nerve could have been involved, leading to the unilateral left VC palsy, and the blurred vision could have been an early manifestation of sclera involvement. Additionally, negative ANCA, normal kidney function and lack of nasal and respiratory involvement make an alternative diagnosis of ANCA vasculitis unlikely.

A key learning point from this case is that the presenting symptoms of RPC of worsening dysphagia and ear pain and erythema might more usually be attributed to worsening of SSc, with auricular manifestations resembling Raynaud’s and vasculopathy of the helix of the ear, and dysphagia as a possible consequence of progressive oesophageal involvement. Moreover, ninth and tenth cranial nerve involvement in RPC has rarely been described.^6,16^ In this patient, misdiagnosis would have led to a diagnostic work-up and treatment management that would have been greatly different with a potentially serious impact on the outcome, since RPC can be a life-threatening disease but usually responds well to steroids unlike SSc. Nevertheless, the acuteness of onset of dysphagia in this patient coupled with the neurologic findings prompted the line of clinical reasoning and investigations which clinched the diagnosis. Therefore, this case highlights the challenging features of RPC, even more so in the background of a previous multisystemic connective tissue disease such as SSc, in which signs of an overlap disease can be more easily missed. This also reminds physicians of the importance of performing a thorough clinical evaluation and considering all possible differential diagnoses when new symptoms arise in the setting of a previously established disease.

This case also resonates with the ongoing debate about vasculopathy *versus* vasculitis in SSc. Vasculopathy is a main and well-established disease mechanism in SSc.^7,15^ On the contrary, so far, there is no irrefutable evidence that a vasculitic process can be an intrinsic part of the SSc pathogenesis in certain patients, since inflammation seems to be mainly perivascular.^7,17^ However, histological specimens with simultaneous features of both vasculitis and vasculopathy in SSc patients have been reported.^7^ On the other hand, overlap of SSc with a systemic vasculitis, namely with ANCA vasculitis, is well recognised, albeit rare.^7,18^ In fact, it has been shown that several HLA haplotypes from the DQ and DP loci can be shared between SSc and ANCA vasculitis.^18^ Interestingly, RPC primarily affects cartilaginous tissues but can both present with manifestations resulting from an underlying vasculitic mechanism, or overlap with other vasculitic entities such as ANCA vasculitis.^2,4^–^6,9^ Therefore, more than just a coincidence, a shared genetic network of autoimmunity predisposing for SSc, inflammation of cartilaginous structures and vasculitis could be the hidden answer to this patient’s presentation and the SSc and RPC overlap.
